# Supplementary material for: The Relationships between Dopaminergic, Glutamatergic, and Cognitive Functioning in 22q11.2 Deletion Syndrome: A Cross-Sectional, Multimodal 1H-MRS and 18F-Fallypride PET Study
Source: Genes (Basel). 2022 Sep 19;13(9):1672. doi: 10.3390/genes13091672 (PMC9498700; doi:10.3390/genes13091672)
Supplement: Supplementary file 1 [file genes-13-01672-s001.zip › genes-1906795-supplementary.pdf]

Supplementary Online Material

van Hooijdonk CFM, Tse DHY, Roosenschoon J, Ceccarini J, Booij J, van Amelsvoort TAMJ, Vingerhoets C. The relationships between dopaminergic, glutamatergic, and cognitive functioning in 22q11.2 deletion syndrome: a cross-sectional multimodal ^{1}H-MRS and ^{18}F-fallypride PET study.

Table of contents:

Table S1. Overview of CANTAB domains and tasks

Table S2. Association between dopaminergic and glutamatergic functioning in 22q11DS

Table S3. Association between cognitive functioning and dopamine $D_{2/3}$ receptor availability in 22q11DS

Table S1. *Overview of CANTAB domains and tasks*[a]

**Domain**	**Task**
Visual learning and memory	Paired Associate Learning (PAL)
Verbal learning and memory	Verbal Recognition Memory (VRM)
Working memory	Spatial Working Memory (SWM)
Attention and vigilance	Rapid Visual Processing (RVP)
Processing speed	Reaction Time (RTI)
Reasoning and problem solving	One Touch Stockings of Cambridge (OTS)
Social cognition	Emotion Recognition Test (ERT)

[a]A detailed description can be found online http://www.cambridgecognition.com/cantab/cognitive-tests/ .

Table S2. *Association between dopaminergic and glutamatergic functioning in 22q11DS*[a]

	ACC glutamate	ACC glutamine	ACC Glx	Striatum glutamate	Striatum glutamine	Striatum Glx
BP_{ND} [^{18}F]fallypride CNC (left)	r = -0.32	r = -0.16	r = -0.25	r = 0.37	r = 0.55	r = 0.32
	p = 0.37	p = 0.65	p = 0.49	p = 0.29	p = 0.13	p = 0.37
BP_{ND} [^{18}F]fallypride CNC (right)	r = -0.15	r = -0.30	r = -0.50	r = 0.15	r = 0.00	r = 0.01
	p = 0.68	p = 0.41	p = 0.14	p = 0.68	p = 1.00	p = 0.99
BP_{ND} [^{18}F]fallypride putamen (left)	r = -0.58	r = -0.44	r = -0.44	r = -0.29	r = 0.17	r = -0.20
	p = 0.08	p = 0.20	p = 0.20	p = 0.43	p = 0.67	p = 0.58
BP_{ND} [^{18}F]fallypride putamen (right)	r = -0.41	r = -0.26	r = -0.42	r = 0.07	r = 0.12	r = 0.24
	p = 0.24	p = 0.47	p = 0.23	p = 0.86	p = 0.77	p = 0.51
BP_{ND} [^{18}F]fallypride VST (left)	r = -0.10	r = 0.06	r = 0.03	r = 0.33	r = -0.33	r = 0.27
	p = 0.78	p = 0.88	p = 0.93	p = 0.35	p = 0.38	p = 0.45

BP_{ND} [^{18}F]fallypride VST (right)	r = -0.39	r = -0.36	r = -0.44	r = 0.31	r = -0.10	r = 0.22
	p = 0.26	p = 0.31	p = 0.20	p = 0.39	p = 0.80	p = 0.53

Abbreviations: ACC, anterior cingulate cortex; BP_{ND}, binding potential; CNC, caudate nucleus; Glx, glutamate plus glutamine; VST, ventral striatum.
[a]Only correlations with $p < 0.0125$ were deemed statistically significant (0.05/(2 [^{1}H-MRS metabolites] x 2 [^{1}H-MRS brain regions]); Bonferroni correction).

Table S3. Association between cognitive functioning and dopamine $D_{2/3}$ receptor availability in 22q11DS[a]

	BP_{ND} [18F]fallypride CNC (left)	BP_{ND} [18F]fallypride CNC (right)	BP_{ND} [18F]fallypride putamen (left)	BP_{ND} [18F]fallypride putamen (right)	BP_{ND} [18F]fallypride VST (left)	BP_{ND} [18F]fallypride VST (right)
Visual memory	r = 0.06	r = 0.16	r = 0.18	r = 0.19	r = -0.35	r = -0.23
	p = 0.88	p = 0.65	p = 0.63	p = 0.60	p = 0.33	p = 0.53
Verbal memory	r = 0.07	r = 0.07	r = -0.13	r = -0.26	r =-0.70	r = -0.49
	p = 0.86	p = 0.86	p = 0.73	p = 0.47	**p = 0.03**	p = 0.15
Working memory	r = -0.27	r = -0.03	r = 0.12	r = 0.18	r = -0.30	r = -0.32
	p = 0.46	p = 0.93	p = 0.75	p = 0.63	p = 0.40	p = 0.37
Attention[b]	r = 0.08	r = 0.30	r = -0.05	r = -0.13	r = -0.60	r = -0.22
	p = 0.83	p = 0.43	p = 0.90	p = 0.73	p = 0.09	p = 0.58
Processing speed	r = -0.14	r = 0.24	r = -0.36	r = -0.13	r = -0.03	r = -0.02
	p = 0.70	p = 0.51	p = 0.31	p = 0.73	p = 0.93	p = 0.96

Executive functioning	r = -0.48	r = -0.31	r = -0.25	r = -0.09	r = -0.26	r = -0.34
	p = 0.16	p = 0.38	p = 0.49	p = 0.81	p = 0.47	p = 0.34
Social cognition	r = -0.12	r = 0.03	r = -0.04	r = 0.13	r = -0.60	r = -0.46
	p = 0.75	p = 0.93	p = 0.92	p = 0.73	p = 0.07	p = 0.18
Composite score	r = -0.08	r = 0.06	r = -0.07	r = -0.13	r = -0.54	r = -0.41
	p = 0.83	p = 0.88	p = 0.86	p = 0.73	p = 0.11	p = 0.24
FSIQ	r = 0.10	r = 0.27	r = 0.06	r = 0.27	r = 0.09	r = 0.21
	p = 0.78	p = 0.44	p = 0.87	p = 0.46	p = 0.80	p = 0.57

Significant results before Bonferroni correction are bold.
Abbreviations: ACC, anterior cingulate cortex; BP_{ND}, binding potential; CNC, caudate nucleus; VST, ventral striatum.
[a]Only correlations with p < 0.00555 were deemed statistically significant (0.05/9 [7 cognitive domains, composite score, and FSIQ]; Bonferroni correction).
[b]One 22q11DS subject was excluded from the analysis which focused on the cognitive domain attention due to an extreme value.
